# Multi-assessed green sustainable chromatographic resolution of nicotine and caffeine; application to in-vitro release from a new quick mist mouth spray co-formula

**DOI:** 10.1186/s13065-024-01306-z

**Published:** 2024-10-15

**Authors:** Yomna A. Salem, Ahmed Emad F. Abbas, Amgad E. Salem, Aya A. Abdella, Amal A. El-Masry

**Affiliations:** 1 Department of Pharmaceutical Chemistry, Faculty of Pharmacy, Sinai University, Kantara Branch, Ismailia, 41636 Egypt; 2 Analytical Chemistry Department, Faculty of Pharmacy, October 6 University, 6 October City, Giza, 12585 Egypt; 3 Department of Pharmaceutics, Faculty of Pharmacy, Mansoura University, Mansoura, 35516 Egypt; 4 Department of Pharmaceutical Analytical Chemistry, Faculty of Pharmacy, Tanta University, Elguish Street (Medical Campus), Tanta, 31527 Egypt; 5 Department of Medicinal Chemistry, Faculty of Pharmacy, Mansoura University, Mansoura, 35516 Egypt

**Keywords:** Nicotine, Caffeine, Green chromatographic method, Quick mist spray, *in-vitro* release, Saliva, Green assessment

## Abstract

The availability of well-established analytical methods is crucial to cope with the fast-ongoing research for the development of new drug delivery formulations. In this work, a rapid highly green chromatographic method was developed for the simultaneous determination of nicotine (NIC) and caffeine (CAF) to be applied for an in-vitro release study from a newly prepared quick mist mouth spray co-formula (QMS), as a complementary synergistic fast-onset relief of cravings during smoking cessation. The chromatographic resolution was accomplished on a cyano column using isocratically delivered (1.0 mL/ min) glycerol: orthophosphoric acid (OPA) (0.2 M) adjusted to pH 3.0 using 0.05 M triethylamine (5:95, v/v) and UV detection at 260 nm. Well resolved peaks of NIC and CAF were eluted at 2.1 and 3.9 min (Rs = 5.64), with linear responses between 0.1 and 20.0 µg/mL and 0.2–40.0 µg/mL, and detection limits of 0.03 and 0.07 µg/mL for NIC and CAF, respectively. The developed method showed good analytical performance (accuracy, precision, robustness, and selectivity) as well as superiority in practicality and ecological profile compared to reported methods applying GAPI, analytical eco-scale, AGREE, BAGI, and whiteness metric tool. The developed method was successfully applied for NIC and CAF determination in their pharmaceutical preparations, and artificial saliva with no significant differences from reported method results (F-test and *t*-test). Moreover, an *in-vitro* release study of NIC and CAF from QMS was performed employing the developed method that revealed diffusion-controlled release, compared to mixed diffusion/ polymer chain relaxation for marketed single component formulation, showing the superiority of QMS in reducing drug level fluctuations of NIC and CAF and improving their bioavailability.

**Graphical Abstract:**

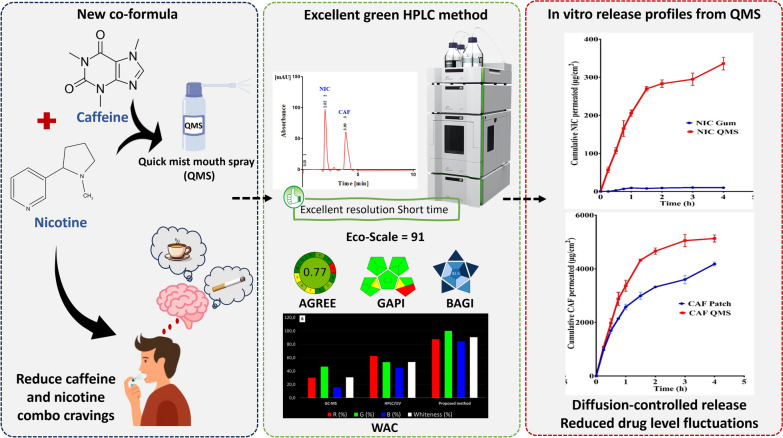

**Supplementary Information:**

The online version contains supplementary material available at 10.1186/s13065-024-01306-z.

## Introduction

Nicotine (NIC) and caffeine (CAF) are central nervous system (CNS) stimulants that act on the brain causing several effects. NIC, 3-[(2S)-1-methylpyrrolidin-2yl] pyridine [1], is a naturally produced alkaloid in the nightshade family of plants (predominantly in tobacco and *Duboisia hopwoodii*) commonly used as an anti-anxiety and CNS stimulant. It exerts its pharmacological action by stimulating specific nicotinic and dopaminergic receptors in the brain, thereby enhancing their release. Currently, more than 1.2 billion people worldwide consume different tobacco products that result in nicotine addiction [2]. As a pharmaceutical drug, it has been incorporated in various dosage forms such as transdermal patches, sublingual and inhalers, as well as lozenges, to be used for tobacco cessation programs, thus easing the transition from cigarette smoking to complete abstinence [3]. Several methods were reported in the literature for NIC determination, in different samples such as extracts, electronic cigarettes, gums, patches, plasma, saliva, and sweet, including chromatographic [4–10], UV–Vis spectrophotometric [11, 12], spectrofluorimetric [13], electrochemical [14, 15] methods.

Caffeine (CAF), 1,3,7-trimethylpurine-2,6-dione [1], a natural stimulant of the methylxanthine class, is used for a variety of purposes, including premature newborns with respiratory troubles, pain relief, and drowsiness. It exerts its action by inhibiting phosphodiesterase enzymes in skeletal muscle and adipose tissues and promoting lipolysis via the activation of hormone-sensitive lipases [16]. With repeated caffeine dosing, paraxanthine may contribute to development of tolerance and withdrawal symptoms. Coffee affects the brain's nicotine receptors, which might explain the coffee-cigarette morning combo familiar to smokers. Therefore, co-administration of NIC and CAF has been reported also during smoking cessation to help alleviating these withdrawal symptoms [17]. Moreover, CAF augments the effect of NIC replacement therapy to reduce weight gain after smoking cessation [18].

Regarding the simultaneous determination of CAF and NIC, very few methods are reported in literature, including HPLC/ UV [19, 20], and gas chromatography-tandem mass spectrometry [21], which almost use environmentally hazardous organic solvents, such as methanol and acetonitrile. Also, these methods employ either extensive extraction prior to chromatographic separation [19], applies mobile phase with 80% buffer that highly increased backpressure of instrument [20] or requires expensive instrumentation [21]. Additionally, the reported methods suffer from long run time which exceeded 22 min for the greenest one [20]. The availability of well-established analytical methods is crucial to cope with the fast-ongoing research for the development of new drug delivery formulations. Since analysis is an essential act to evaluate drug release from the developed formula, the chosen method needs to fulfill some criteria including accuracy, selectivity, cost-effectiveness, rapidness, and minimal environmental impact. Liquid chromatography has been recognized as the most efficient and first choice technique that enables the fast, selective resolution and subsequent determination of mixtures in a wide variety of samples.

Nevertheless, since the different chromatographic procedures involve the use of huge amounts of organic modifiers, they could have significant harmful effects on the environment [22]. Therefore, scientists have bestowed their efforts to make the chromatographic separation greener. This could be attempted by discovering new non-toxic organic modifiers to replace the well-known hazardous solvents such as methanol [23, 24]. A wide variety of organic modifiers have been introduced as greener alternatives such as propylene carbonate-ethanol mixtures [25], ethyl acetate [26], ethyl lactate [27], and glycerol [28–30]. Nevertheless, the practical application of such greener solvents is still limited and hence, this work is devoted to broadening the scope of application of glycerol as green mobile phase modifier.

Glycerol is a natural tri-hydroxyl alcohol comes from cheap available sources including palm trees and tallow rendered fat animals. Besides, glycerol is a chemically inert, non-volatile, highly stable environmentally friendly organic modifier. Replacing acetonitrile or methanol in various chromatographic applications with glycerol renders the developed method greener, highly efficient, cost saving and completely nontoxic to both human and environment [26].

The aim of this work was to develop a new green HPLC–UV method, utilizing glycerol as organic modifier, to be utilized for the determination of NIC and CAF in their newly co-formulated quick mist mouth spray (QMS) along with an in-vitro release study. Moreover, the proposed HPLC–UV method was successfully applied for the determination of NIC and CAF in their marketed dosage forms and saliva. Method green analytical profile, practicality and analytical efficiency were evaluated employing both green analytical procedure index (GAPI) tool [31], analytical Eco-scale [32], Analytical GREEnness (AGREE) calculator [33], Blue Applicability Grade Index (BAGI) [34], and whiteness [35] metrics showing its superiority over the reported methods, as it eradicated the use of harmful organic solvents, and ensured its efficiency and practicality. It is worth noting that our developed method affords efficient separation (Rs = 5.64) in a short run time (< 5 min). Thus, it can be adopted for their routine analysis in research and quality control labs.

## Materials and methods

### Chemicals

Liquid NIC (purity 99%) was supplied by Wuhan Vanz Pharm Inc (Wuhan, China). CAF (purity 99%) reference substance was provided by Novartis (Basel, Switzerland). HPLC grade Triethylamine, methanol and acetonitrile were supplied by Sigma-Aldrich (Steinheim, Germany). Analytical reagent grade glycerol and Orthophosphoric acid (OPA) were obtained from Prolabo (Paris, France). Nicorette^®^ gum and Nercafix^®^ oro-dispeersible films, labeled to contain 4 mg, 40mg of NIC and CAF, respectively were purchased from the local market. Highly purified deionized water was used throughout the study. Potassium chloride, sodium chloride, calcium chloride anhydrous, sodium hydroxide, and monosodium phosphate anhydrous were obtained from the Egyptian pharmaceutical manufacturer EL-Nasr (ADWIC), which were utilized for preparation of artificial saliva.

### Apparatus

A Perkin Elmer TM Series 200 Chromatograph equipped with a Rheodyne injector valve with a 20 μL loop and a UV/VIS detector was used for performing chromatographic separations. Data collection and processing was attempted using Total Chrom Workstation (Massachusetts, USA). Shim-pack cyano column (150 mm × 4.6 mm with 5.0 µm particle size), CLC Shim-Pack C_8_ column (250 × 4.6 mm, 5 mm particle size), Shimadzu, Kyoto, Japan, and knaur C_18_ column (250 × 4.6 mm, 5 mm particle size), Agela Technologies, Wilmington, USA, were tried. pH measurements were accomplished using a Consort NV P-901 pH –Meter (NV P-901, Belgium, (JENWAY 3540, USA)). *In-vitro* permeation was studied using the modified Franz’s diffusion cells (D.K. Scientific, Ahmedabad, India).

### Chromatographic conditions

**Fig. 1:**
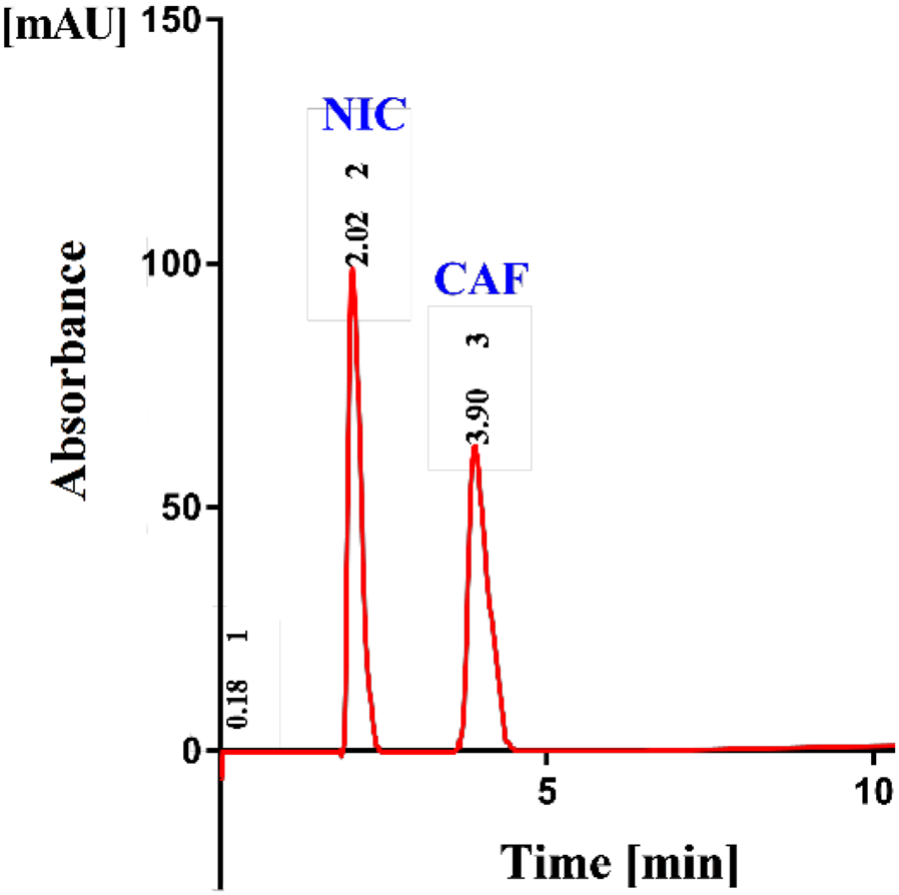
Typical chromatogram showing the well resolved peaks of NIC 10.0 µg/mL, 2.02 min), CAF (20.0 µg /mL, 3.9 min.) in synthetic mixture

The chromatographic separation of NIC and CAF was attempted on a shim-pack cyano column (150 mm × 4.6 mm with 5.0 µm particle size) with column temperature maintained at 40ºC. A mobile phase composed of glycerol: OPA (0.2 M) adjusted to pH 3.0 using diluted solution of 0.05 M triethylamine (5:95, v/v) was delivered at a flow rate of 1.0 mL/min with UV detection at 260 nm and injection volume of 20 μL. The mobile phase was sonicated for 30 min, filtered using 0.45 µm membrane filter (Millipore, Ireland), and then degassed using Merck solvent L-7612 degasser. The mobile phase was stable when stored in refrigerator at 4 ֯C for 2 weeks. The developed chromatogram, as given in (Fig. 1), illustrated the optimum separation of NIC and CAF in their synthetic mixture.

### Analytical procedures and calibration graphs

Stock solution (200 μg/mL) of NIC was prepared by direct diluting 5.051 mL of pure liquid standard NIC (purity, 99% = 99 mg in 100 mL) with methanol in a 25-mL volumetric flask. Stock solution (500 μg/mL) of CAF was prepared by dissolving 50.0 mg of CAF in deionized water in a 100-mL volumetric flask. The stock solutions were stored in refrigerator at 4 °C in amber container. The concentration ranges of 0.1- 20.0 and 0.2- 40.0 μg/mL of NIC and CAF, respectively, were prepared by dilution in a series of 10-mL measuring flasks. The solutions were made up to total volumes mobile phase before being injected into the HPLC system. The solutions were injected triplicate (20 μL) applying the previously mentioned chromatographic conditions (Sect. "Chromatographic conditions"). Calibration graphs were constructed by plotting the resulting peak area against the corresponding drug concentration (μg/mL). Binary mixture was prepared in the ratio of 1:2 of NIC: CAF, respectively and analyzed triplicate (Sect. "Chromatographic conditions").

### Fabrication and drug content determination of the prepared QMS

The composition of 0.4% (w/w) NIC and 4.0% (w/w) CAF QMS are shown in Table S1. QMS was prepared by solubilizing the NIC and CAF in deionized water (DW) and ethanol with constant stirring at moderate speed (100 rpm) for 1 h at 40 °C to complete solubility and the other components are added. The total weight was adjusted to 100.0 g with DW. Each 1.0 g of QMS contains 4.0 mg NIC and 40.0 mg CAF.

For drug content determination, an accurate weight (1.0 g) of the NIC/CAF QMS containing 4.0 mg NIC and 40 mg CAF, Nercafix® oro-dispeersible films containing 40.0 mg CAF, and Nicorette® gum containing 4.0 mg NIC were transferred into separated 100-mL stoppered volumetric flasks. About half a volume of flasks was filled with methanol (50 mL), vortexed for 30 min, sonicated for 30 min, to extract the drugs, and then completed to final marks with methanol. The contents were filtered through Millipore filter (0.45 µm) before being analyzed using the developed method (Sect. "Chromatographic conditions"). The mean % drug content and ± SD were calculated [36].

### Preparation of artificial saliva

Artificial saliva (pH = 6.5) was prepared following the formula reported by Liu et al. [37] and Butt et al. [38] to imitate normal oral conditions. The artificial saliva was prepared by dissolving KCl (0.4 g/L), NaCl (0.4 g/L), CaCl_2_ (0.9 g/L), NaH_2_PO_3_ (buffer component) (0.7 g/L), and the pH was adjusted using NaOH.

### In-vitro permeation study

The *in-vitro* release study was carried out on 1.0 g NIC/CAF QMS containing 4.0 mg NIC and 40.0 mg CAF, Nercafix® oro-dispeersible films contain 40.0 mg CAF, and Nicorette® gum containing 4.0 mg NIC. The medium used was 0.05 M phosphate buffer (pH 7.4), maintained at 37°C ± 0.5°C. Cellophane dialysis membrane was used as a permeation barrier. Samples were collected at predetermined time intervals (0.25, 0.5, 0.75, 1, 2, 3, and 4 h.). Samples were analyzed for drug content employing the developed HPLC method (see Sect. "Chromatographic conditions"). All permeation studies were three replicates for each formulation.

Linear regression analysis for the drug release data was done to determine the drug release kinetics and mechanism. The in vitro release results were fitted to various kinetic models (zero-order, first order, diffusion-controlled release mechanism (Higuchi model), and Korsmeyer–Peppas model) to determine the mechanism of the drug release as indicated by the higher correlation coefficient and coefficient of determination (R^2^).

## Results and discussion

In this study, to cope with the fast-ongoing research for the development of new drug delivery formulations serving both pharmaceutical industry and easily applicable in quality control labs, a new highly green HPLC–UV method, utilizing glycerol as organic modifier, was first introduced for the determination of NIC and CAF in their newly co-formulated quick mist mouth spray (QMS) and an inclusive in-vitro release study. Moreover, the proposed HPLC–UV method was successfully applied for the determination of NIC and CAF in their marketed dosage forms and saliva.

The developed method is a more environmentally friendly option compared to other complicated chromatographic procedures since it requires few-to-no tedious sample preparations and considered to be straightforward, automated, and highly reproducible. The organic modifier used throughout the study is glycerol. Replacing acetonitrile or methanol in various chromatographic applications with glycerol renders the developed method greener, highly efficient, cost saving and completely nontoxic to both human and environment.

### Method development and optimization

NIC and CAF are hydrophilic compounds possessing too close Log *P* values (1.17, and -0.07, respectively) [39]. Moreover, NIC (Fig. S1) has two basic nitrogen groups in its chemical structure, pka_1_ = 3.12, pka_2_ = 8.02, and hence it exists in three forms depending on the pH of the solution [40]. On the other hand, CAF (Fig. S1) contains more than two basic nitrogen groups in its chemical structure (pka_1_ = 10.4, pka_2_ = 0.7) [41]. This renders their separation using green chromatographic procedures challenging. Recently, Habib et al. [29, 30] has introduced glycerol as a new green organic modifier for chromatographic resolution and determination of drugs having low or moderate Log *P*. Therefore, glycerol was chosen as mobile phase modifier in this work.

Efficiency is the critical aspect affecting the separation in liquid chromatography because it affects both peak shape and resolution [42, 43]. Compared with other hazardous organic modifiers, high polarity of glycerol enabled optimum regulation of mobile phase elution strength and separation of both ionized and non-ionized analytes. Thus, it was applied efficiently for quantitative separation of NIC and CAF using CN column. The different factors affecting NIC and CAF separation were studied. These factors include pH, concentration of glycerol, concentration of OPA, temperature, and flow rate.

Table S2 shows the calculated chromatographic performance parameters, including the number of theoretical plates (NTP), at each of the studied factors.

#### Selection of column

Several stationary phase columns were tried including Shim-pack cyano column and CLC Shim-Pack C_8_ column in different dimensions were separately examined. The tried C8 column displayed asymmetric non resolved solvent and CAF peaks, and the run time exceeded 10 min. Replacing C8 colum by Cyano column, it gave result to symmetrical peaks with an excellent resolution for CAF and NIC and reasonable retention time within only 4 min. The best resolution was achieved using Shim-pack cyano column (150 mm × 4.6 mm with 5.0 µm particle size).

#### Selection of detection wavelength

Moreover, different wavelengths were tried, including 210, 254, 260 and 280 nm. The detection wavelength was chosen to be 260 nm as it enabled the simultaneous estimation of both drugs without any interference with high sensitivity.

#### Effect of pH

For resolution of these basic drugs, the pH of the mobile phase critically affects selectivity, retention, resolution, and efficiency. The ionization behavior of NIC and CAF is almost similar, so the effect of pH on efficiency was studied. The pH of the mobile phase was investigated over the range 2.5 to 5.5. As NIC and CAF are completely ionized over the investigated pH range, their retention times were negligibly affected when the pH value was raised to 6.0. However, regarding NIC, higher efficiency was achieved at pH 3.0, as shown in Fig. S2. This can be attributed to the predominance of fully ionized form as the pH is maintained at 3.0. At higher pH values, NIC would exist in two forms leading to peak tailing, poor efficiency, and poor resolution (Table S2).

##### Effect of glycerol concentration

It is reported that increasing glycerol concentration in the mobile phase shortens the retention time of hydrophilic compounds. However, this increase is limited by the glycerol induced back pressure and pump capacity. Accordingly, different concentrations of glycerol (5 to 8% (v/v)) were studied in relation to the number of theoretical plates (Fig. S3), 5% (v/v) was chosen as the optimum (Table S2).

##### Effect of OPA concentration

As presented in Table S2, different OPA concentrations ranging from 0.1 to 0.3 M were studied. As shown in Fig. S4, the optimum OPA concentration was found to be 0.2 M as it provided the highest sensitivity, best separation efficiency, reasonable run time, and maximum selectivity.

##### Effect of column temperature

Column temperature critically affects the rate of mass transfer between mobile phase and stationary phase in addition to mobile phase viscosity and the glycerol induced back pressure. Therefore, column temperature was raised (from 35 to 45 ֯ C) to increase the NTP of both drugs (Fig. S5). It could be noted that 40 ֯C could achieve highest efficiency and resolution and lowest back pressure (Table S2).

##### Effect of Flow rate

Flow rate is reported among the factors that can help overcoming the limitations of glycerol as organic modifier. Accordingly, the effect of flow rate (0.8–1.2 mL/min) on the peak separation of the studied drugs was examined (Table S2). The A flow rate of 1.0 mL/min could achieve best resolution with maximum NTP, as shown in Fig. S6.

### Method validation

The developed green HPLC–UV method was validated as per ICH Q2 (R1) guidelines [44].

#### Linearity and limits of detection (LOD) and quantitation (LOQ)

Regarding linearity, the method exhibited excellent linearity (r = 0.9999) over concentration ranges of 0.1- 20.0 µg/mL and 0.2–40.0 µg/mL, for NIC and CAF, respectively. Moreover, the validity of the constructed calibration graphs was evident by small values of residual standard deviation and % error, as presented in Table 1. The limit of detection (LOD) was found to be 0.03 and 0.7 µg/mL for NIC and CAF, respectively.

**Table 1:** Regression parameters for the determination of NIC and CAF using the proposed green chromatographic method

Parameter	NIC	CAF
Linearity range (μg/mL)	0.1–20.0	0.2–40.0
Correlation coefficient (r)	0.9999	0.9999
Slope ± SD^a^	317.13 ± 3.17	24.65 ± 0.49
Intercept ± SD^a^	31.58 ± 0.33	18.82 ± 0.03
LOD^b^ (μg/mL)	0.03	0.07
SD^a^ of residuals	6.61	1.02
% RSD^c^	1.24	1.25
% Error	0.44	0.44

^a^ Standard deviation

^b^ Limit of detection

^c^ Relative standard deviation

#### Accuracy and precision

Moreover, the accuracy and precision of the developed method were tested using triplicate determinations of a synthetic mixture containing both drugs at three different concentrations 5.0, 10.0 and 15.0 µg/mL and at 10.0, 20.0 and 30.0 µg/mL of NIC and CAF, respectively. The mixtures were analyzed within the same day (for evaluation of intraday precision) and three different days (for inter-day precision evaluation) (Table 2). Furthermore, the obtained percentage recovery values in addition to the small values of % Error demonstrated method accuracy. RSD < 2% for both intraday and inter-day precision indicated good precision (Table 2).

**Table 2:** Recovery results for evaluation of accuracy and precision for the determination of NIC and CAF by the proposed green chromatographic method

Parameter		NIC concentration (mg/mL)			CAF concentration (mg/mL)		
		0.5	1.0	1.5	1.0	2.0	3.0
Intra-day (n = 3)	Mean	100.84	99.40	100.50	102.71	99.40	101.40
	± SD	1.53	0.76	0.95	0.15	0.76	1.31
	% RSD	1.51	0.77	0.95	0.16	0.77	1.29
	% Error	0.87	0.44	0.55	0.09	0.44	0.74
Inter-day (n = 3)	Mean	99.50	100.18	100.90	99.02	100.67	99.23
	± SD	1.13	1.06	1.66	0.67	1.93	1.15
	% RSD	1.14	1.06	1.65	0.68	1.92	1.16
	% Error	0.66	0.61	0.95	0.39	1.11	0.67

^*^Each result is the mean recovery of three separate determinations

Method accuracy was further approved by comparing three different concentrating selected within the linearity ranges for both drugs in Synthetic Mixture (Table 3) and different dosage forms (QMS, gums and patches) (Table 4) separately with reference method [19]. The excellent percentage recoveries (%R) between 98 and 102% for repeated measurements displayed outstanding accuracy, as shown in Tables 3 and 4.

**Table 3:** Assay results for the determination of NIC and CAF in their synthetic mixture

Conc. taken (μg/mL)		% Recovery*			
		Synthetic mixture		Reference method [19]	
NIC	CAF	NIC	CAF	NIC	CAF
0.5	1.0	100.11	100.18	99.52	99.4
1.0	2.0	100.24	100.07	99.99	100.25
1.5	3.0	99,91	99.89	99.89	99.52
Mean ± SD		100.09 ± 0.17	100.05 ± 0.148	99.55 ± 0.58	99.72 ± 0.46
%Error		0.1	0.09	0.33	0.27
t- value		1.55 (2.78)	1.18 (2.78)		
*F-*value		11.99 (19)	9.61 (19)

**Table 4:** Assay results for the determination of NIC and CAF in QMS, Nicorette gum and Nercafix® oro-dispeersible films

Conc. taken (μg/mL)		% Recovery*					
		QMS		Gum	Patches	Reference Method [19]	
NIC	CAF	NIC	CAF	NIC	CAF	NIC	CAF
0.5	5.0	100.11	100.18	98.12	100.58	99.13	101.57
1.0	10.0	100.24	100.09	98.83	99.12	98.55	100.81
1.5	15.0	99.94	99.89	98.08	99.57	99.86	100.37
Mean ± SD		100.09 ± 0.17	100.05 ± 0.148	98.87 ± 0.23	99.85 ± 0.91	99.18 ± 0.66	100.92 ± 0.61
%Error		0.1	0.09	0.13	0.52	0.38	0.35
t- value		2.32 (2.78)	2.39 (2.78)	0.76 (2.78)	2.09 (2.78)		
*F*-value		15.69 (19)	16.73 (19)	8.28 (19)	1.52 (19)		

^*^Each result is the mean recovery of three separate determinations

#### Selectivity

**Fig. 2:**
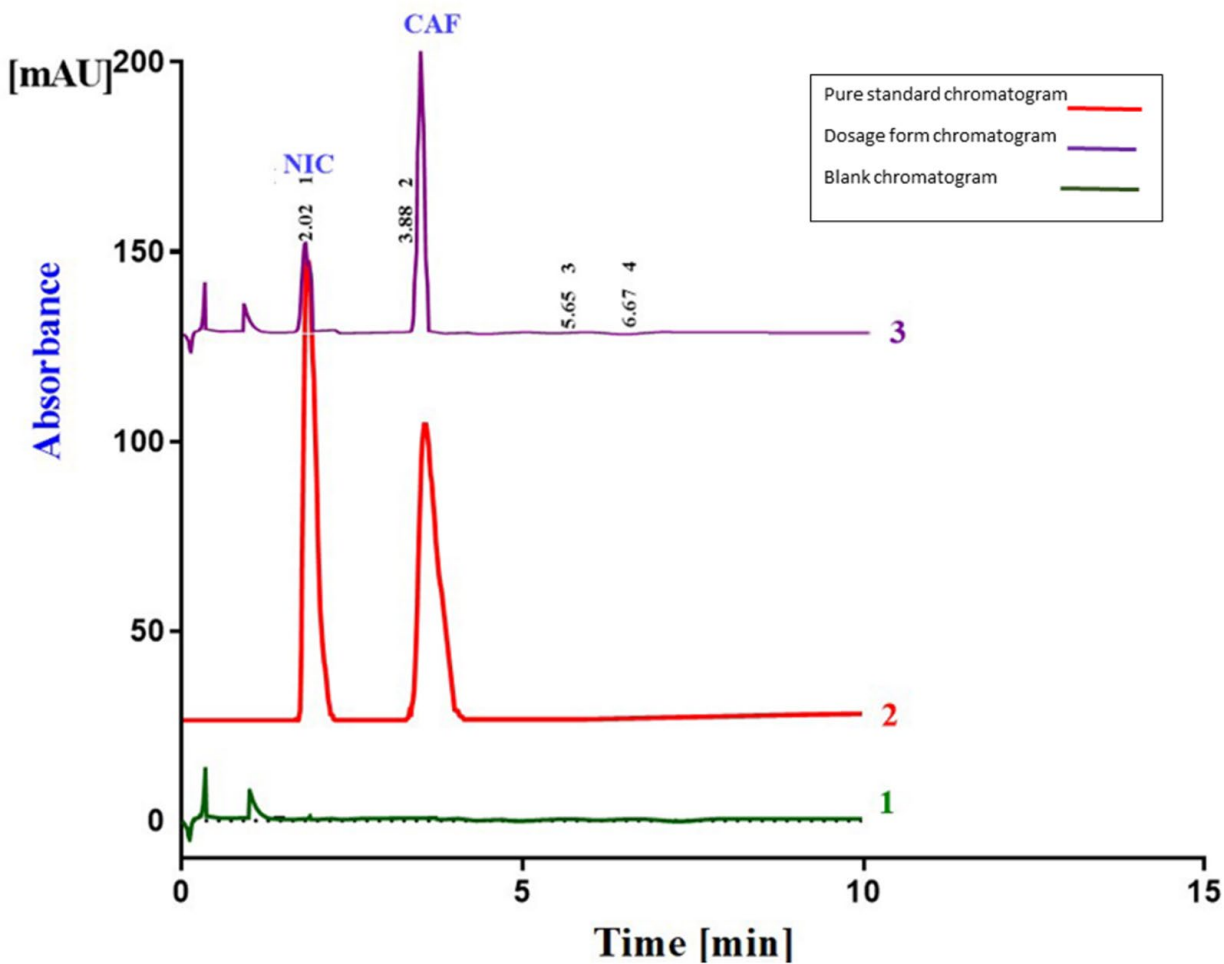
Typical chromatograms for the separation of NIC (10.0µg/mL, 2.05 min), CAF (20.0 µg /mL, 3.9 min) in pure form, dosage form (2.5µg/mL, 2.05 min), CAF (25.0 µg /mL, 3.9 min) and blank. Chromatographic system: shim-pack cyano column (150 mm × 4.6 mm with 5.0 µm particle size, pressure adjusted to 1960 psi, pressure adjusted to 1960 psi) Mobile phase mobile phase consisting of Glycerol: 0.2 M OPA (5: 95% v/v), pH 2.5 flow rate, 1.0 mL/min, UV detection at 260 nm; column temperature, 40 degrees Celsius

Method selectivity was demonstrated by the observed well resolved NIC and CAF peaks in dosage form compared to blank showing method capability for the simultaneous determination of the studied drugs without any interferences (Fig. 2). Moreover, no interference was observed from the matrix components during the analysis of studied drugs by the developed glycerol-based separation method indicating its specificity as confirmed in a combined chromatogram of pure standard of NIC and CAF, their dosage forms and their blank (Fig. 2). This was further confirmed by consistency of NIC and CAF recovery results from lab prepared QMS with a reported HPLC–UV comparison method (Table 4).

#### Robustness

Furthermore, method robustness was evaluated by investigating the effect of deliberate intended modifications in the optimized chromatographic conditions on the measured response (AUC). The studied factors included pH (3.0 ± 0.1), glycerol concentration (5.0 ± 0.4% v/v), column temperature (40.0 ± 2.0 °C), and flow rate (1.0 ± 0.1) (Table S3). Accordingly, the robustness of the proposed method was evident by the unaffected recovery results for both drugs.

#### System suitability

To evaluate system performance, system suitability parameters like the number of theoretical plates (N), mass distribution ratio (DM), relative retention **(α),** and resolution (Rs) were all investigated as system appropriateness criteria. The efficiency of the chromatographic work system was indicated by acceptable system suitability parameters as presented in Table S2.

### Sample application

#### Determination of NIC and CAF in their dosage forms

The developed method was successfully applied for the determination of NIC in Nicorette® gum and CAF in Nercafix® oro-dispeersible films (Table 4). Moreover, a new QMS fixed dose combination containing NIC and CAF was assayed using the method was applied for the simultaneous determination of the studied drugs in with acceptable accuracy and precision as indicated by % Error and %RSD, respectively.

##### Application for the determination of NIC and CAF in the artificial saliva

Furthermore, the developed method was applied successfully for simultaneous determination of NIC and CAF in synthetic saliva. The results were accurate and precise as indicated by lower values of % Error and %RSD, respectively (Table 5).

**Table 5:** Application for the determination of NIC and CAF in artificial saliva

Conc. taken (μg/mL)		% Recovery *	
NIC	CAF	NIC	CAF
0.25	2.5	101.99	99.63
1.25	12.5	100.49	99.06
2.5	25	102.12	102.05
Mean ± RSD		101.53 ± 0.89	100.25 ± 1.58
%Error		0.52	0.92

^*^Each result is the mean recovery of three separate determinations

##### Application for *in-vitro* permeation study of NIC and CAF from QMS

**Fig. 3:**
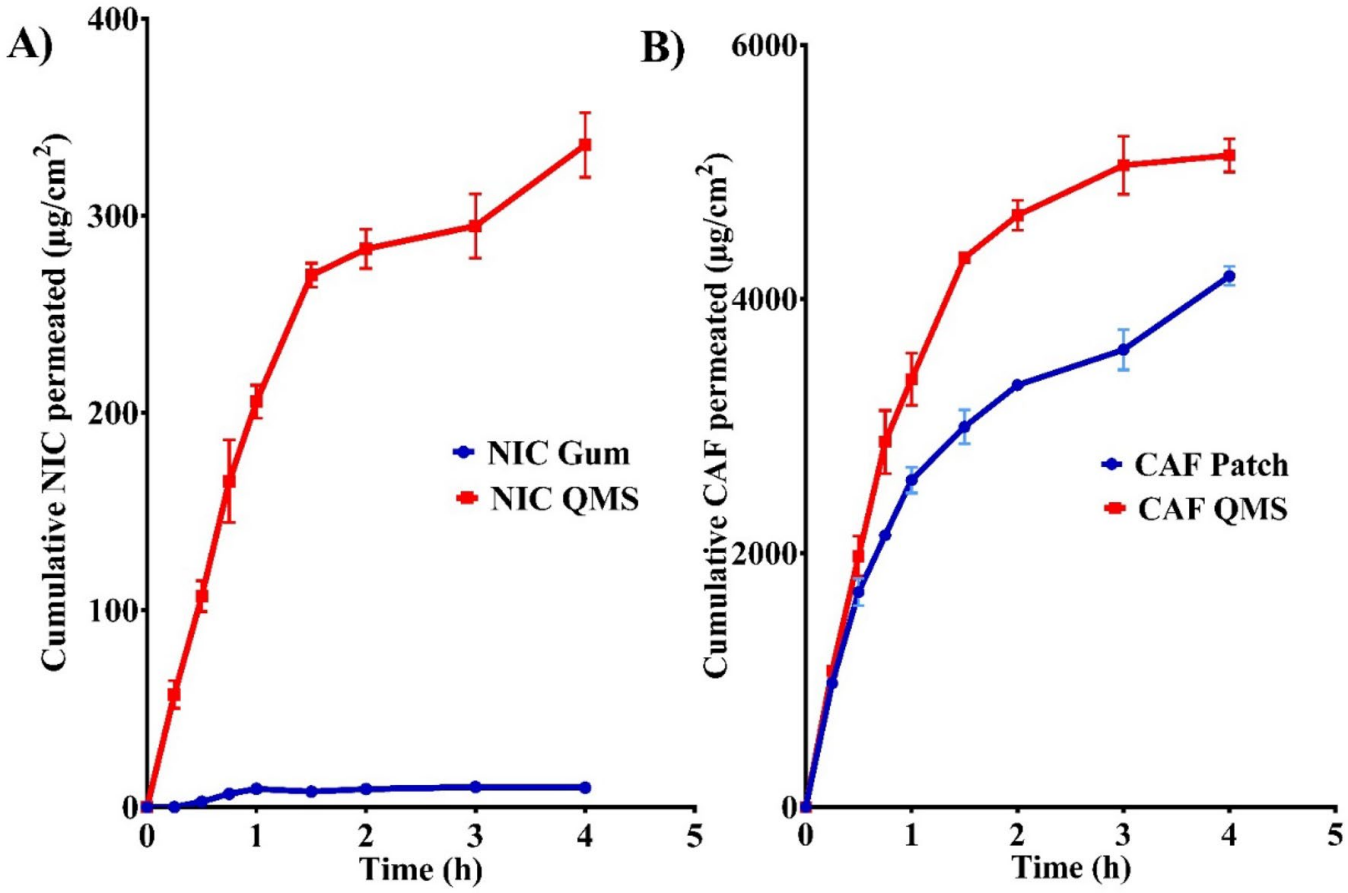
In vitro release profiles of NIC from QMS and gum (**A**) and CAF from QMS and patch (**B**) through cellophane membrane

The NIC/CAF QMS containing 4.0 mg NIC, and 40.0 mg CAF showed significantly higher release than that observed with control formula (Nercafix^®^ oro-dispeersible films contain 40.0 mg CAF, and Nicorette^®^ gum containing 4 mg NIC) (p < 0.05) (Fig. 3). The flux of NIC from the QMS and Nicorette^®^ gum was found to be 77.62 ± 8.041, and 2.462 ± 0.4017 µg/cm^2^.h, respectively which is 31.5 folds higher than that of the control. The flux of CAF from the QMS was found to be 1196 ± 135.10 µg/cm^2^.h, which is 1.33 folds higher than that of the control (Nercafix^®^ oro-dispeersible film) (898.7 ± 85.90 µg/cm^2^.h). These results showed that the type of base could greatly influence the flux of the drug. Besides, the promised efficacy in smoking cessation and excellent release behavior of newly formulated QMS in industrial field compared to other marketed formulations.

The data of kinetic analysis are represented in Table S4. The in vitro release data for the tested formulations were best explained by Higuchi model (diffusion-controlled mechanism) for most tested formulations and the plots showed the highest linearity among the models investigated. This suggests that the release of NIC and CAF occurred mainly by diffusion [45]. Accordingly, the exponent (n) values, Kinetic analysis of the release of NIC from QMS and CAF from QMS and patch indicated that the formulations have non-Fickian (n ≥ 0.45) diffusion predominated suggesting that a combination of diffusion and polymer chain relaxation [46]. While the release of NIC from gum indicated that the formulation has a super case II transport. Super Case-II release is the drug transport mechanism associated with stresses and state transition in hydrophilic polymers which swell in water or biological fluids [47]. The advantages of using diffusion-controlled release are the flexibility to tune rate of release via regulation of polymeric carrier physicochemical properties, ease to achieve and lower consumption of expenses [45].

### Evaluation of method greenness

**Fig. 4:**
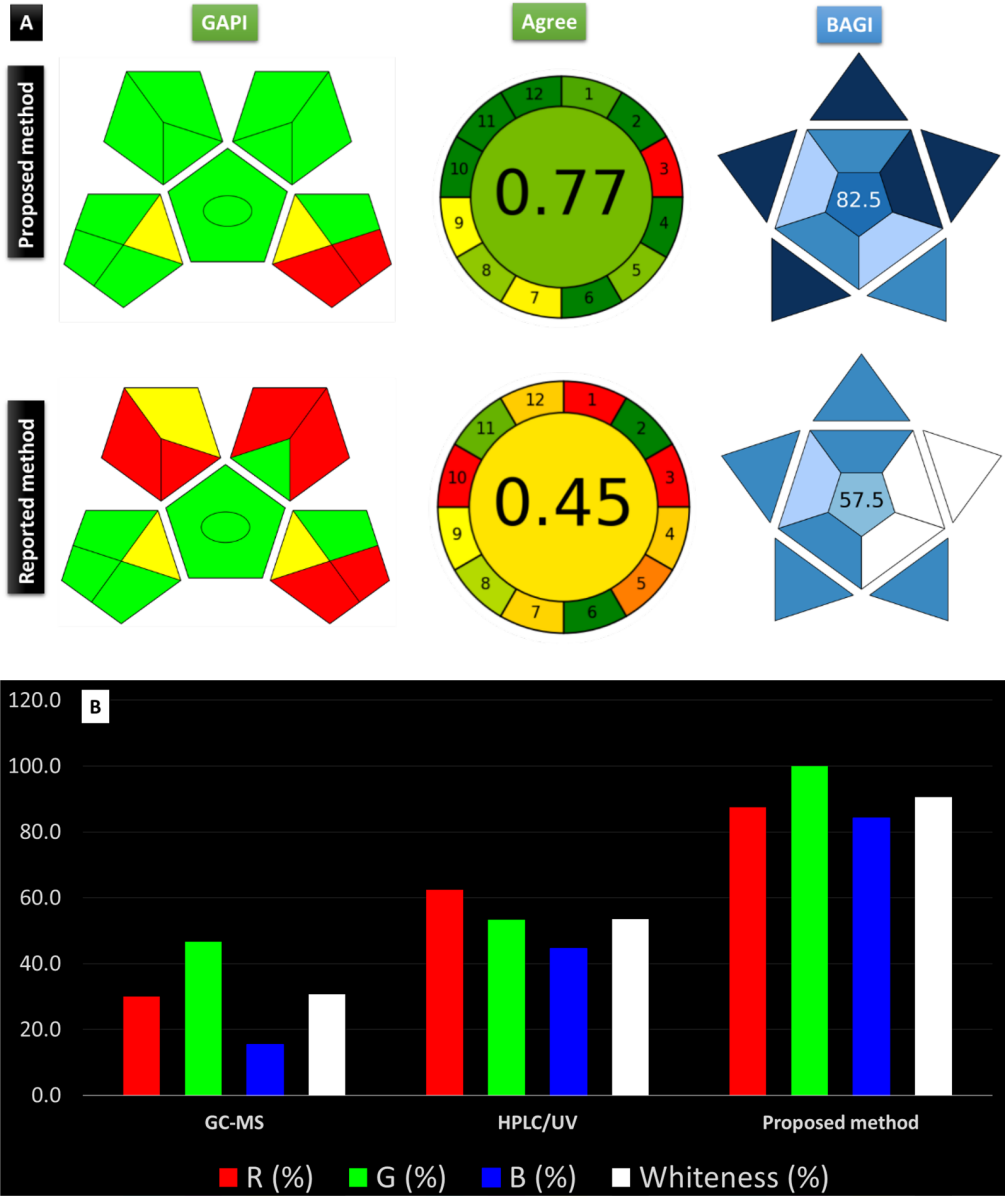
**A** Greenness evaluation of the developed method according to GAPI, AGREE, BAGI compared to the reported chromatographic method [19], **B** Whiteness of the proposed method compared to reported methods [19, 21]

**Fig. 5:**
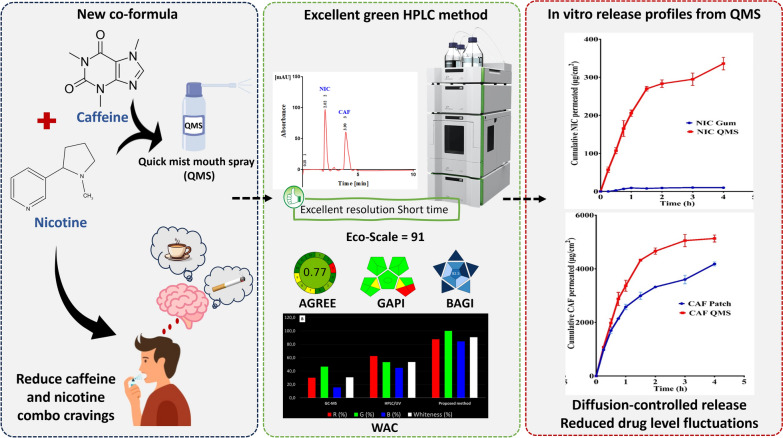
Graphical abstract

Different tools were applied to comprehensively evaluate the ecological impacts of the method compared to those previously reported for simultaneous determination of NIC and CAF utilizing three different metric tools including analytical eco-scale, GAPI, and AGREE. Regarding analytical eco scale, it applies penalty points to calculate eco-score (100-pp) that reflect method associated environmental hazards [32]. The new method achieved an eco-score of 91 compared to the reported method [19] (eco-score of 79) (Table S5). In addition, GAPI tool, used to point out the potential source of hazard in the form of pentagrams evaluating each step in the analytical procedure using specific color codes, was utilized [31]. The developed method showed less hazards than the reported HPLC–UV method [19] as it utilizes bio based organic modifier and produced less waste. Moreover, AGREE calculator was applied to compare the performance of both methods as it considers safety and method throughput [33]. As shown in Fig. 4A, the developed method showed advantages in safety, minimization of energy consumption and method throughput. Thus, the developed method can be safely and efficiently applied for routine QC work due to short run time which enables the analysis of larger number of samples per hour. Despite the demonstrated excellent green performance for the developed chromatographic method, this does not necessarily indicate method applicability and suitability for the intended use. Therefore, BAGI metric tool was applied to evaluate the practical applicability of the developed method for QC labs [34]. BAGI tool uses an asteroid pictogram together with a respective score which reflects method performance in the form of ten practicality attributes. The developed method exhibited a run time of 5 min resulting in sample throughput up to 12 sample/ hour, no enrichment was necessary, and sample volume was 20 μl. A BAGI score of 82.5 demonstrated method practicality (Fig. 4A). To ensure method functionality, method whiteness was calculated using WAC calculator [35]. WAC uses a quantitative parameter called whiteness to evaluate method performance as a function of 12 principles including greenness (G1-G4), efficiency (R1-R4), and practicality/ economics (B1-B4). The whiteness was calculated for the developed method compared to reported GC/MS [21] and HPLC/UV methods [19] (Fig. 4B). The developed method demonstrated clear superiority indicated by whiteness score of 90.6%. As shown in Fig. 4B, the enhancement in method greenness did not compromise its functionality. Thus, the developed method could efficiently overcome most of the limitations encountered by methods available in literature in terms of mobile phase hazardousness, run time, and analysis throughput (Table S6). A graphical abstract provides a visual summary of the key findings of a study, making it easier to quickly grasp the main points and significance of the research (Fig. 5).

## Conclusion

This study introduced the development, optimization, and validation (ICH Q2 R1 guidelines) of a rapid, affordable, and green liquid chromatographic method for simultaneous determination of co-administered drugs; NIC and CAF. Using glycerol as a green mobile phase modifier is replacing other commonly hazardous solvents in HPLC and having several advantages in chromatography including biodegradability, low UV cut-off point, and lowest degree of instability and explosiveness. Thus, it is completely harmless to both environment and human. The developed method enabled the determination of the studied drugs in their separate dosage forms as well as synthetic mixtures and artificial saliva. Furthermore, developed method was applied to study the *in-vitro* release of both drugs from a newly formulated QMS and the satisfactory results serves pharmaceutical industry via promoting new dosage form with rapid adsorption and a faster onset of relief to smoking urge compared to other marketed pharmaceuticals. The green performance of the developed method was confirmed using GAPI, AGREE and analytical Eco-scale. The developed methods excellently met the criteria of green analytical method model including reduced use of hazardous solvents or reagents, diminished energy consumption, decreased waste generation, and increased method throughput and number of samples per hour. In addition, practical applicability was demonstrated by BAGI score of 82.5 and 90% whiteness score. Accordingly, the developed green chromatographic method could be used for routine analysis of both drugs in quality control labs.

## Supplementary Information

Supplementary file 1.

## Data Availability

The datasets used and/or analyzed during the current study are available from the corresponding author on reasonable request.

## Abbreviations

HPLC: High performance liquid chromatographic method
NIC: Nicotine
CAF: Caffeine
QMS: Quick mist mouth spray
DW: Deionized water
OPA: Orthophosphoric acid
GAPI: Green analytical procedure index
AGREE: Analytical GREEnness
BAGI: Blue Applicability Grade Index
ICH: International council for harmonization
% RSD: Percentage relative standard deviation
LOD: Limit of detection
LOQ: Limit of quantification
