# Supplementary material for: Multi-assessed green sustainable chromatographic resolution of nicotine and caffeine; application to in-vitro release from a new quick mist mouth spray co-formula
Source: BMC Chem. 2024 Oct 15;18(1):200. doi: 10.1186/s13065-024-01306-z (PMC11476497; doi:10.1186/s13065-024-01306-z)
Supplement: Supplementary file 1 [file 13065_2024_1306_MOESM1_ESM.pdf]

Supplementary materials

Multi-assessed green sustainable chromatographic resolution of nicotine and caffeine; application to in-vitro release from a new quick mist mouth spray co-formula

Yomna A. Salem[1*], Ahmed Emad F. Abbas[2], Amgad E. Salem[3], Aya A. Abdella[4], Amal A. El-Masry[5]

1. Yomna A. Salem (***Corresponding author**)

   Department of Pharmaceutical Chemistry, Faculty of Pharmacy, Sinai University – Kantara Branch, Ismailia 41636, Egypt.

   Email: *Yomna.abdulaziz@su.edu.eg & yomnasalem26@gmail.com*

   Tel. +201023622361

2. Ahmed Emad F. Abbas

   Department of Pharmaceutical Analytical Chemistry, Faculty of Pharmacy, October 6 University, 6 October City, Giza 12585, Egypt.

   Email: *ahmed.emad.pha@o6u.edu.eg*

3. Amgad E. Salem

   Department of Pharmaceutics, Faculty of Pharmacy, Mansoura University, Mansoura, 35516, Egypt.

   Email: amgadsalem@mans.edu.eg

4. Aya A. Abdella, PhD

   Department of Pharmaceutical Analytical Chemistry, Faculty of Pharmacy, Tanta University, Elguish street (Medical Campus), 31527 Tanta, Egypt

   Email: Aya.atef.86@gmail.com

5. Amal A. El-Masry

   Department of Medicinal Chemistry, Faculty of Pharmacy, Mansoura University, Mansoura, 35516, Egypt.

   Email: dr.Amal90@mans.edu.eg

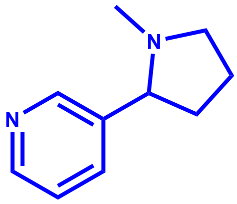

Nicotine (NIC)

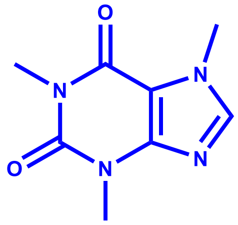

Caffeine (CAF)

Fig. S1. Structural formula of nicotine (NIC) and caffeine (CAF)

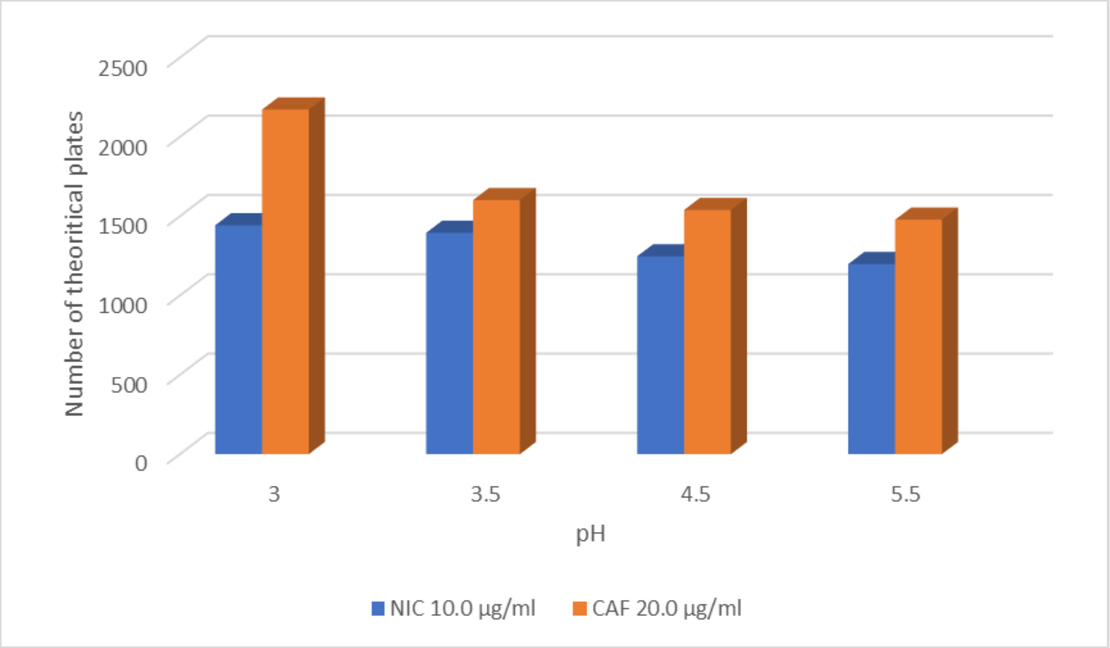

Fig. S2. Effect of different pH on the number of theoretical plates of NIC 10.0 µg/ml and CAF 20.0 µg/ml using mobile phase consisting of Glycerol: 0.2 M OPA, (5: 95% v/v). Flow rate, 1.0 mL/min, UV detection at 260 nm; column temperature, 40 degrees Celsius.

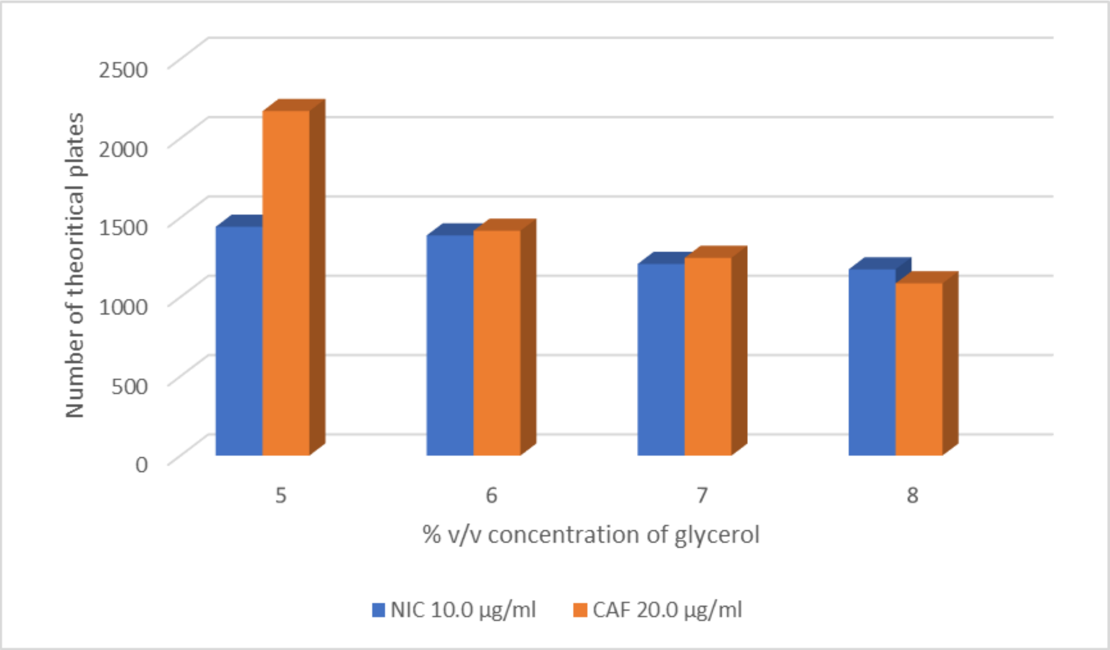

Fig. S3. Effect of different % v/v concentration of glycerol on the number of theoretical plates of NIC 10.0 µg/ml and CAF 20.0 µg/ml using a mobile phase containing 0.2 M OPA pH 3.0, Flow rate, 1.0 mL/min, column temperature, 40 degrees Celsius.

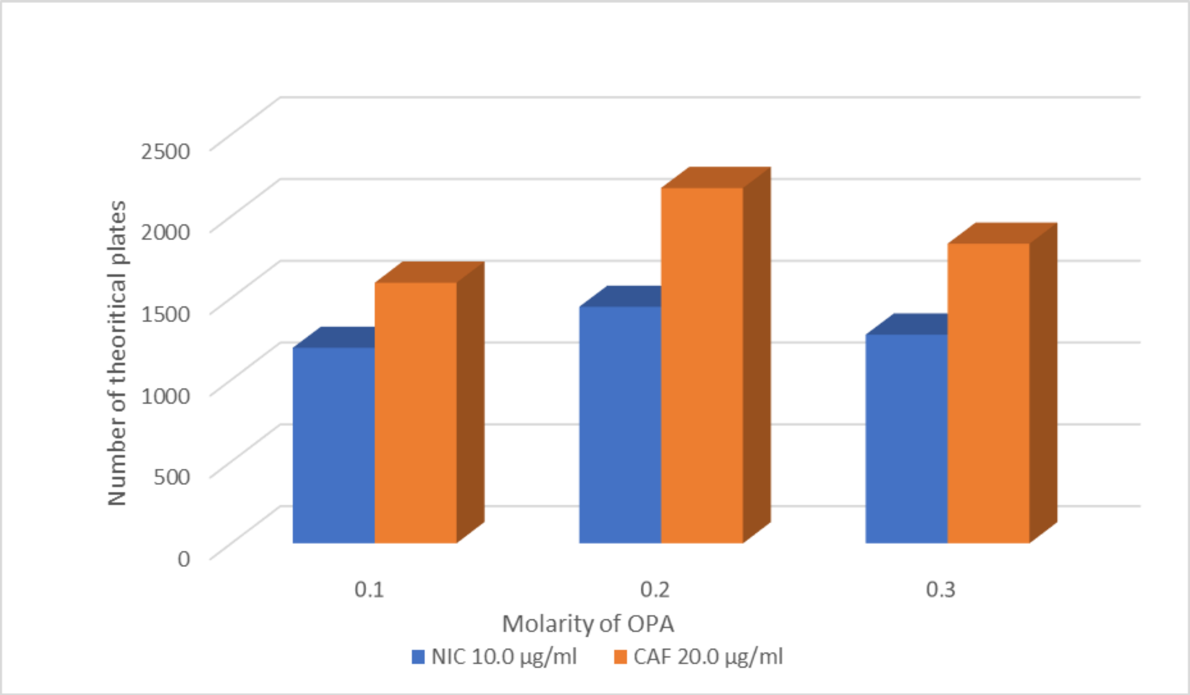

Fig. S4. Effect of different molarity of OPA on the number of theoretical plates of NIC 10.0 µg/ml and CAF 20.0 µg/ml using a mobile phase mobile phase consisting of Glycerol: 0.2 M OPA, (5: 95% v/v), pH 3.0, Flow rate, 1.0 mL/min, column temperature, 40 degrees Celsius.

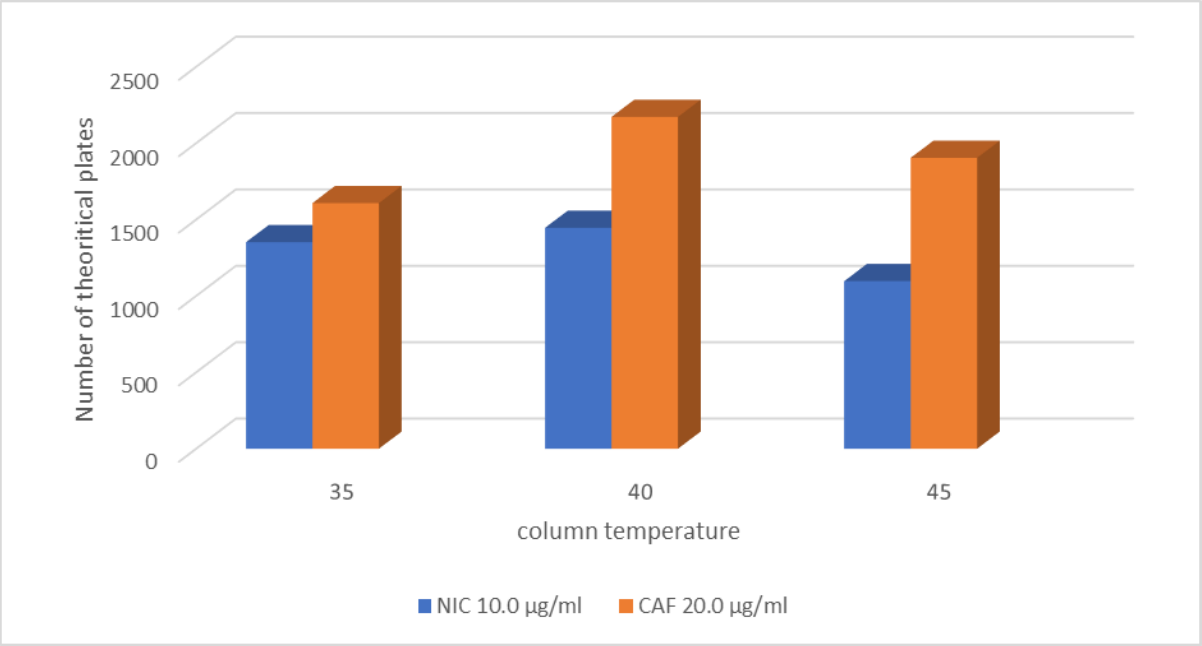

Fig. S5. Effect of column temperature in Celsius degree on the number of theoretical plates of NIC 10.0 µg/ml and CAF20.0 µg/ml using mobile phase consisting of Glycerol: 0.2 M OPA, (5: 95% v/v), pH 3.0. Flow rate, 1.0 mL/min, UV detection at 260 nm.

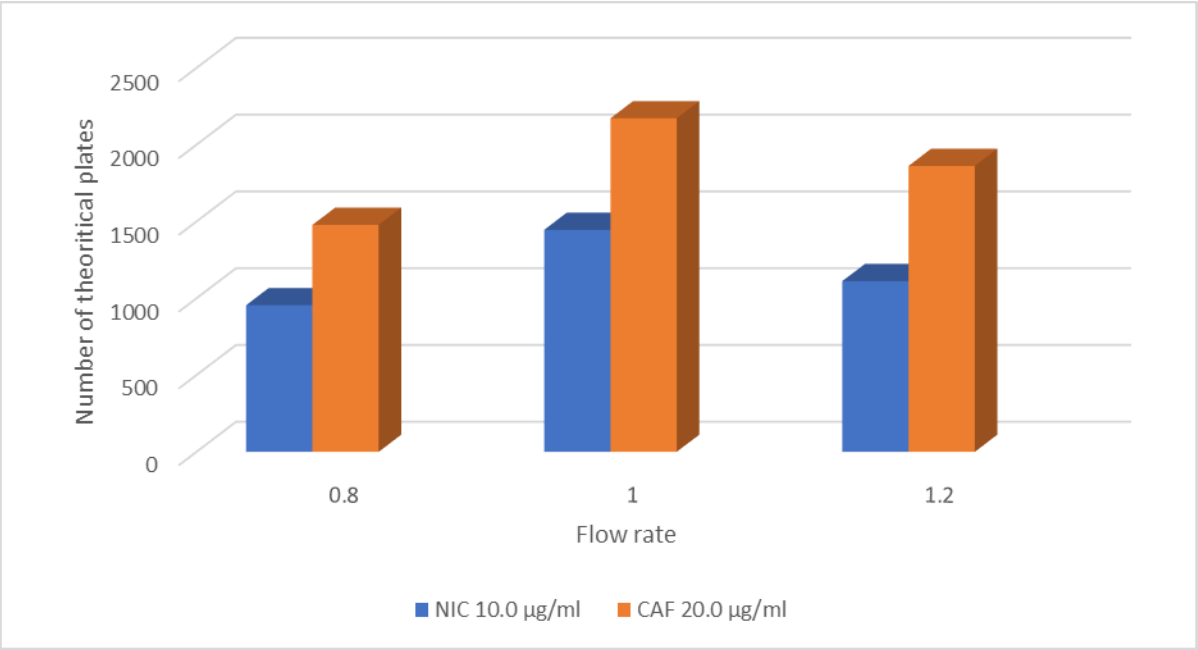

Fig. S6. Effect of flow rate on the number of theoretical plates of NIC 10.0 µg/ml and CAF 20.0 µg/ml using mobile phase consisting of Glycerol: 0.2 M OPA, (5: 95% v/v), pH 3.0. UV detection at 260 nm, column temperature, 40 degrees Celsius.

Table S1. Composition of 0.4% (w/w) NIC and 4% (w/w) CAF QMS

Ingredients	Amount %(w/w)
**NIC**	0.4
**CAF**	4
**Ethanol**	15
**Propylene glycol**	10
**Butylated hydroxytoluene**	0.1
**Distilled Water to**	100

Table S2: Optimization of the chromatographic parameters for separation of mixture of NIC and CAF by the proposed green chromatographic method.

Parameter		No. of theoretical plates (N)		Mass distribution ratio (Dm)		Resolution (Rs)	Pressure	Relative retention (α)
		NIC	CAF	NIC	CAF	Rs	p.	α
pH	3.0	1445.64	2171.9	1.33	2.6	5.64	1960	1.95
	3.5	1394.7	1601.1	0.98	2.54	4.86	2143	1.51
	4.5	1246.5	1538.8	0.84	2.46	4.88	2613	1.57
	5.5	1198.1	1477.9	0.78	2.5	5.11	2876	1.61
Percentage Ratio of [Glyerol: 0.2 M OPA]	(5:95)	1445.64	2171.9	1.33	2.6	5.64	1960	1.95
	(6:94)	1387.9	1418.2	1.08	2.78	4.91	2589	2.58
	(7:93)	1207.7	1246.5	0.833	2.5	4.66	2789	2.9
	(8:92)	1173.9	1085.5	0.769	2.23	4.15	3098	3.1
Molarity of OPA	0.1	1194.74	1592.8	1.21	2.54	4.83	1960	2.1
	0.2	1445.64	2171.9	1.33	2.6	5.64	1960	1.55
	0.3	1275.3	1831.4	0.833	2.5	4.93	2148	2.8
Temp.	35	1352	1608.1	1.08	2.83	4.88	2780	2.61
	40	1445.64	2171.9	1.33	2.6	5.64	1960	1.55
	45	1097.3	1903.5	1.37	2.8	5.59	1890	2.03
Flow rate	0.8	955.2	1477.9	0.73	2.26	4.62	1597	3.09
	1.0	1445.64	2171.9	1.33	2.6	5.64	1960	2.77
	1.2	1111.8	1862	1.12	3.12	5.33	2464	1.55

Where: Number of theoretical plates (N) = $5.45(t_R/ W_{h/2})^2$,

- $W_{h/2}$ is the half peak width
- Resolution (R) = $2\ \Delta\ t_R/W_1 + W_2$,
- $W_1$and W_2 are the peaks width of the two components measured at their bases.
- Relative retention (α) = Dm_2/ Dm_1,
  Mass distribution ratio (Dm) = t_R-t_m/ t_m, t_R is time of retention of the measured substance measured from the injection point and t_m is the retention time of a non-retained marker.

Table S3: Robustness results for determination of NIC and CAF by the proposed green chromatographic method.

Parameter	Value	NIC			CAF		
		% Recovery	Mean % recovery ± SD	%RSD	% Recovery	Mean % recovery ± SD	%RSD
**pH**	2.9	99.52	100.49 ± 0.84	0.84	102.54	101.03 ± 1.41	1.40
	3.0	101.02			99.76		
	3.1	100.94			100.78		
**Glycerol concentration %**	4.6	98.42	99.19 ± 0.92	0.93	97.51	98.28 ± 0.67	0.68
	5.0	98.94			98.74		
	5.4	100.21			98.58		
**Temperature (˚C)**	38	98.51	100.34 ± 1.65	1.64	100.81	101.87± 1.03	1.01
	40	100.78			101.92		
	42	101.73			102.87		
**Flow rate**	0.9	98.24	98.78 ±1.6	1.62	99.52	99.67 ± 1.01	1.01
	1.0	100.58			98.74		
	1.1	97.53			100.75		

Table S4. Kinetic modeling of release data from NIC/CAF QMS and trade marketing formulations through cellophane membrane.

Drug	Formula	R^2			Release Order	Korsmeyer et al		Main transport mechanism
		Zero-Order	First Order	Higuchi Model		R^2	n	
NIC	Gum	0.6004	0.3249	0.7647	Diffusion	0.6440	1.26	Super case II
	QMS	0.7885	0.6233	0.9348		0.9078	0.62	Non-Fickian
CAF	Patch	0.8141	0.7058	0.9699		0.9513	0.50	
	QMS	0.7580	0.6178	0.9313		0.9111	0.56	

Table S5. Assessment of green analytical performance of the developed HPLC/UV method using analytical Eco-scale.

Proposed method		Reported method [21]	
**Reagents**	**PPs**	**Reagents**	**PPs**
**Glycerol**	0	Acetonitrile	4
**OPA**	3	Methanol	5
**TEA**	2	Diethyl amine	2
		Acetic acid	4
	Σ5		Σ15
**Instruments**		Instruments	
**HPLC/UV**	1	HPLC/UV	1
**Occupational hazards**	0	Occupational hazards	0
waste	3	waste	5
	Σ4		Σ6
**Total PPs: 9**		**Total PPs: 21**	
**Score: 91 (excellent green analysis)**		**Score: 79 (acceptable green analysis)**	

Table S6. Comparison of the developed green HPLC method to reported methods

Method	Mobile phase	Run time	Analysis throughput	Sample	Limitations	Ref.
**HPLC-UV**	Water: methanol: buffer acetate (pH 4.66): acetonitrile: acetic acid (50:29:20:2:1, v/v)	10 min	6 samples per hour	Meconium	-Utilization of hazardous solvents.<br>-Long runtime.	[19]
**HPLC-UV**	Methanol: buffer (5 mM sodium octane sulphonate, 10 mM citric acid adjusted to pH 5.8 with triethylamine) (20:80)	> 22 min	2 samples per hour	Human milk	-Utilization of hazardous solvents.<br>-Long runtime.<br>- high buffer loads.	[20]
**GC-MS/MS**	Helium 5.0	> 11 min	5 samples per hour	Chocolate	Utilization of highly sophisticated and expensive instrumentation.<br>Tedious sample preparation.	[21]
**HPLC-UV**	Glycerol: orthophosphoric acid (0.2 M) adjusted to pH 3.0 using 0.05 M triethylamine (5:95, v/v)	5 min	12 samples per hour	Dosage forms and artificial saliva.	-	This work
